# Comparison of *Actinobacteria* communities from human‐impacted and pristine karst caves

**DOI:** 10.1002/mbo3.1276

**Published:** 2022-04-07

**Authors:** Andrea Buresova‐Faitova, Jan Kopecky, Marketa Sagova‐Mareckova, Lise Alonso, Florian Vautrin, Yvan Moënne‐Loccoz, Veronica Rodriguez‐Nava

**Affiliations:** ^1^ CNRS, INRAe, VetAgro Sup, UMR 5557 Ecologie Microbienne Université de Lyon, Université Claude Bernard Lyon 1 Villeurbanne France; ^2^ Department of Ecology, Faculty of Science Charles University in Prague Prague 2 Prague Czech Republic; ^3^ Laboratory for Epidemiology and Ecology of Microorganisms Crop Research Institute Praha Czech Republic

**Keywords:** 16S rRNA gene sequencing, *Actinobacteria*, cave anthropization, *hsp*65 sequencing, metabarcoding, Paleolithic cave

## Abstract

*Actinobacteria* are important cave inhabitants, but knowledge of how anthropization and anthropization‐related visual marks affect this community on cave walls is lacking. We compared *Actinobacteria* communities among four French limestone caves (Mouflon, Reille, Rouffignac, and Lascaux) ranging from pristine to anthropized, and within Lascaux Cave between marked (wall visual marks) and unmarked areas in different rooms (Sas‐1, Passage, Apse, and Diaclase). In addition to the 16S rRNA gene marker, 441 bp fragments of the *hsp*65 gene were used and an *hsp*65‐related taxonomic database was constructed for the identification of *Actinobacteria* to the species level by Illumina‐MiSeq analysis. The *hsp*65 marker revealed higher resolution for species and higher richness (99% operational taxonomic units cutoff) versus the 16S rRNA gene; however, more taxa were identified at higher taxonomic ranks. *Actinobacteria* communities varied between Mouflon and Reille caves (both pristine), and Rouffignac and Lascaux (both anthropized). Rouffignac displayed high diversity of *Nocardia*, suggesting human inputs, and Lascaux exhibited high *Mycobacterium* relative abundance, whereas Gaiellales were typical in pristine caves and the Diaclase (least affected area of Lascaux Cave). Within Lascaux, Pseudonocardiaceae dominated on unmarked walls and Streptomycetaceae (especially *Streptomyces mirabilis*) on marked walls, indicating a possible role in mark formation. A new taxonomic database  was developed. Although not all *Actinobacteria* species were represented, the use of the *hsp*65 marker enabled species‐level variations of the *Actinobacteria* community to be documented based on the extent of anthropogenic pressure. This approach proved effective when comparing different limestone caves or specific conditions within one cave.

## Graphical abstract

*hsp*65 Marker as a complement to 16S rRNA gene in *Actinobacteria* community detection with the metabarcoding approach. Some *Actinobacteria* species were detected for the first time in caves.Taxa related to rare opportunistic pathogens detected in anthropized caves. *Streptomyces* were detected in marked areas of Lascaux caves. The in‐house *hsp*65‐based database is available to the scientific community

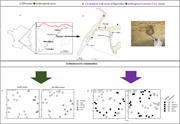

## 1 INTRODUCTION

Limestone caves are isolated habitats that are extremely limited in organic carbon (Barton et al., 2007). They are associated with the development of microbial strategies that enable adaptation to oligotrophic and high calcium conditions. In addition, caves are considered to be extreme environments, as they are oligotrophic, due to the complete darkness, low and constant temperatures, and high humidity (Barton & Jurado, 2007; De Mandal et al., 2017; Ortiz et al., 2014). Some of these caves contain Paleolithic artwork and, consequently, have been frequently visited by tourists for a long time (Alonso et al., 2019; Bontemps et al., 2021; Schabereiter‐Gurtner et al., 2002). Thus, human‐mediated dispersion of endogenous as well as exogenous nutrients and microorganisms inside the caves, together with human metabolism may induce changes in internal microclimatic and microbiological conditions (Hoyos et al., 1998; Mulec, 2014). Microorganisms can be somewhat resilient to changes (Johnston et al., 2012; Tomczyk‐Żak & Zielenkiewicz, 2016). However, community shifts may also occur, which deserves attention if those shifts favor microorganisms with biodeterioration ability (F. Bastian et al., 2010; Bontemps et al., 2021; Fernandez‐Cortes et al., 2011; Sánchez‐Moral et al., 1999) or increase the proportion of species harboring strains with pathogenic potential (Bercea et al., 2019; Neral et al., 2015; Rajput et al., 2012). In such cases, these shifts could represent a threat to cave conservation and the health of visitors.

*Actinobacteria* represent important cave dwellers, and many are potentially pathogenic (Jurado et al., 2010), biotechnologically relevant (Bhullar et al., 2012; Hamedi et al., 2019; Syiemiong & Jha, 2019), and novel (Fang et al., 2017; Jurado et al., 2008; Jurado, Kroppenstedt, et al., 2009; Rangseekaew & Pathom‐Aree, 2019) species have been identified in these environments. *Actinobacteria* can dominate in both anthropized (Gonzalez‐Pimentel et al., 2018) and pristine caves (De Mandal et al., 2017); however, some actinobacterial taxa are specific to one cave type. For example, the autochthonous presence of the Pseudonocardiaceae family has been reported as part of the core microbiome of pristine caves (Lavoie et al., 2017; Porca et al., 2012; Riquelme et al., 2015). However, some species, such as *Pseudonocardia hispaniensis*, have been detected in the moonmilk of a cave associated with urban contamination (Miller et al., 2018), and its presence may be due to its ability to degrade exogenous organic compounds (Lavoie et al., 2017; Porca et al., 2012).

This is also true for the Streptomycetaceae family, which has also been identified among pristine cave inhabitants (Maciejewska et al., 2017). Furthermore, Pseudonocardiaceae and Streptomycetaceae seem to be prominent in pigment‐forming communities on the walls of certain show caves (Cuezva et al., 2012; Porca et al., 2012). In contrast, *Euzebyales* (Gonzalez‐Pimentel et al., 2018), Nocardiaceae (De Mandal et al., 2017), and Gaiellales (Zhu et al., 2019) represent oligotrophic taxa that dominate in pristine caves, most likely because of their ability to degrade complex compounds present in the cave, or to fix CO_2_.

Identification of *Actinobacteria* taxa in these environments has been based on culture‐dependent approaches and 16S rRNA gene sequencing (De Mandal et al., 2017; Yasir, 2018). Culture‐dependent approaches limit community coverage and should, therefore, only be used as a supportive determinant to study their specialized metabolisms (Hamedi et al., 2019; Long et al., 2019). The 16S rRNA gene is the most frequently used marker for bacterial systematics; however, it is unable to resolve individual sequences to the species level (Fox et al., 1992; Hirsch et al., 2010). In addition, identical 16S rRNA gene sequences can be found in different taxa or different 16S rRNA gene sequences within a single species (Větrovský & Baldrian, 2013). However, other (protein‐coding) genes with high sequence polymorphisms used for multilocus sequence analysis (MLSA; i.e., *rpoB*, *gyrB*, and *hsp*65) may help distinguish closely related species from environmental DNA. Thus, they may present greater discriminative power at the species level compared with the 16S rRNA gene (Gao & Gupta, 2012; Takeda et al., 2010; Vos et al., 2012). Thus, using a metabarcoding approach, Aigle et al. (2021) utilized another gene (*tpm*) to evaluate the biodiversity of the bacterial community associated with the degradation of pollutants in anthropized environments at a species level.

In the case of *Actinobacteria*, *hsp*65 encodes a molecular chaperone (a 65‐kDa heat‐shock protein), which, when amplified by specific primers (Telenti et al., 1993), has been used as a molecular marker to identify a large number of isolates (Rodríguez‐Nava et al., 2006, 2007). A single copy of this gene is present in *Actinobacteria* genomes, unlike the 16S rRNA gene marker (Colaco & MacDougall, 2014). Additionally, the length of the amplified *hsp*65 fragment (441 bp) is sufficient for amplicon sequencing. While this marker has been used to assess some *Actinobacteria* groups from environmental samples, it has mostly been used with regard to particular groups, such as *Nocardia* (Vautrin et al., 2021) or *Mycobacterium* (van der Wielen et al., 2013). Therefore, considering these features, the *hsp*65 gene may be a good candidate for detailed assessments of the whole *Actinobacteria* community in extreme environments, such as caves. To make this possible, a reference database encompassing the *Actinobacteria* taxonomic group is required, which should be publicly available for taxonomic identification.

Since the 1960s, the Lascaux Cave has undergone several microbial outgrowth events until recently and has been subjected to (often unsuccessful) chemical treatments, which have resulted in the formation of various visual marks (including stains on walls) that have threatened the Paleolithic artwork (F. Bastian et al., 2010; Bontemps et al., 2021; Martin‐Sanchez et al., 2015). Lascaux was closed to the public in 1963 to ensure cave conservation; despite this, microbial imbalance persisted and surface alterations kept forming, leading to visual marks on wall surfaces (Bontemps et al., 2021). Although the few microbiological studies conducted in this cave have suggested that *Actinobacteria* is a prevalent bacterial group (Alonso et al., 2018, 2019; Martin‐Sanchez et al., 2012), a more complete investigation of the *Actinobacteria* community is lacking, especially at the species level. *Actinobacteria* possess broad metabolic variability, and key functional traits may be related to precise species within the *Actinobacteria*; therefore, we hypothesized that such an assessment would better discriminate *Actinobacteria* communities according to ecological conditions.

The objective of this study was to assess the usefulness of *hsp*65 to complement 16S rRNA gene sequence data using a metabarcoding approach for a deeper, species‐level appraisal of the *Actinobacteria* community in caves, and to assess variation according to cave condition and human disturbance (Mammola, 2019). We compared communities belonging to the phylum *Actinobacteria* between pristine limestone caves (Reille and Mouflon Caves) and anthropized caves (Lascaux and Rouffignac Caves), and within Lascaux Cave between marked (wall visual marks) and unmarked areas in different rooms (i.e., Sas‐1, Passage, Apse, and Diaclase). The second objective was to create an *hsp*65‐based *Actinobacteria* database that is accessible to the scientific community.

## 2 MATERIALS AND METHODS

### 2.1 Study site description and sample collection

**Figure 1:**
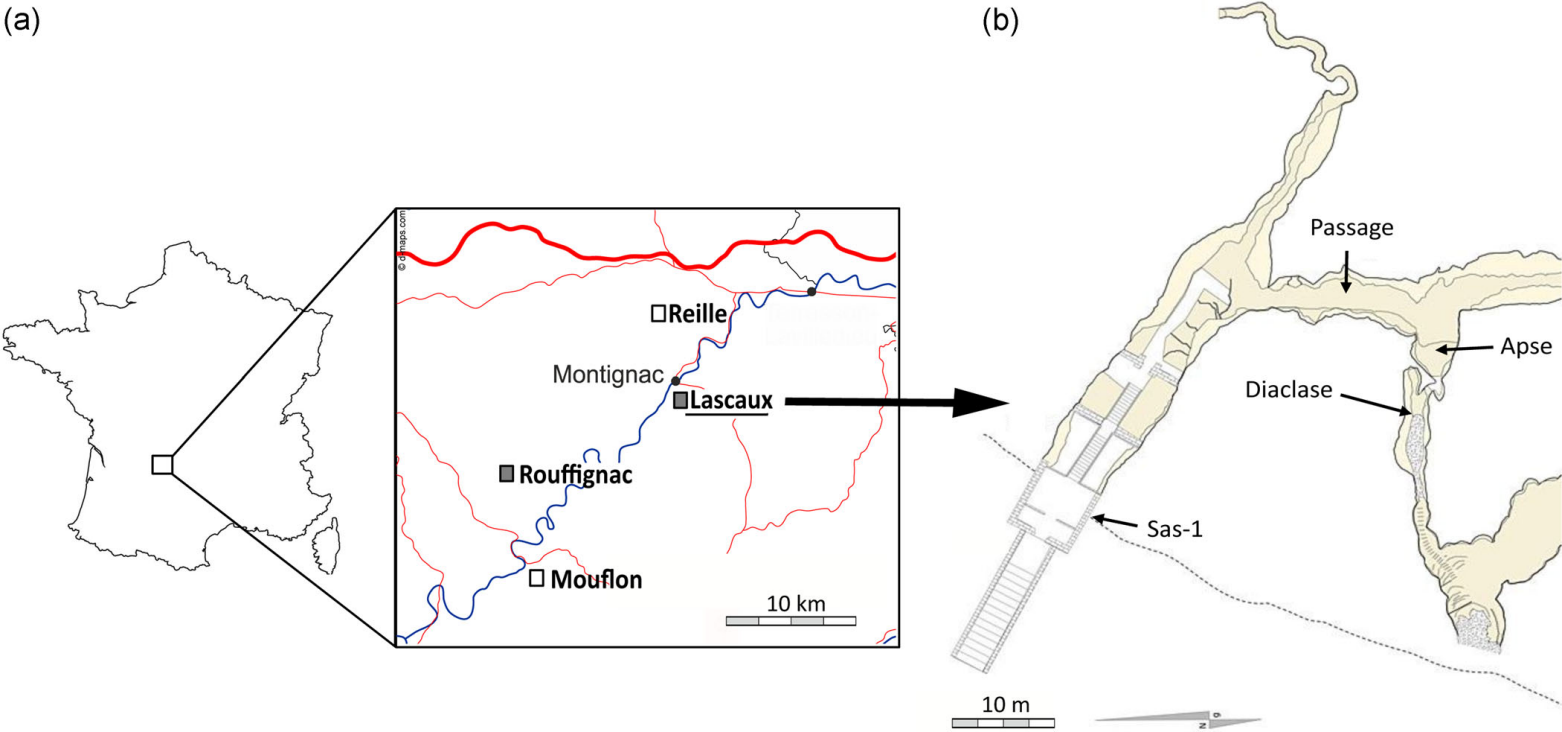
(a) Map of Dordogne area indicating the cave locations (Lascaux, Rouffignac, Reille, and Mouflon; white squares—pristine; gray squares—anthropized). (b) Map of Lascaux Cave (entrance Sas‐1, Passage, Apse, and Diaclase)

Samples were collected in May and June 2016 from the walls of four limestone caves located in the Dordogne department of southwestern France: Lascaux (45°03′13″N, 1°10′12″E, 235 m length), Rouffignac (45°00′31″N, 0°59′16″E, 8000 m length), Reille (45°07′09.5″N, 1°07′11.2″E, 2000 m length), and Mouflon (44°55′N, 1°5′E, 280 m length; Figure 1a).

Lascaux and Rouffignac Caves contain Paleolithic wall paintings and engravings and are listed as UNESCO World Heritage Sites. Both caves are anthropized; Rouffignac has been open to tourist visits since 1959 (with up to 500 visitors per day; Alonso et al., 2019), whereas Lascaux closed after 15 years of tourism (with up to 2000 visitors per day). Lascaux Cave was closed because various visual marks of microbial origin had developed on cave walls. Extensive chemical treatments have been applied to protect the wall paintings from microbial deterioration, which probably triggered further alterations (F. Bastian et al., 2010; De La Rosa et al., 2017; Martin‐Sanchez et al., 2012). Mouflon and Reille Caves remain in a pristine state (Alonso et al., 2019). The entrance of Mouflon Cave was locked shortly after discovery, whereas Reille is occasionally explored by seasoned speleologists.

In Lascaux Cave, samples were taken from the walls of four distinct rooms representing different cave environments that are located progressively farther from the entrance in the following order: Sas‐1, Passage, Apse, and Diaclase (Figure 1b). Sas‐1 is a man‐made, calcareous airlock entrance, and is isolated from the cave interior and exterior by doors. The Passage is a central alley connecting different rooms of Lascaux Cave. In the Passage, samples were taken from two mineral substrates: clay deposits (Passage banks, later referred to as Passage B) and near‐vertical limestone walls (Passage inclined planes, later referred to as Passage IP). In the Apse, samples were collected from limestone inclined planes. The Diaclase is a distinct compartment located below the main part of the cave, which has been least affected by humans. It is separated from the Apse by a trap door, has received very limited biocide treatment, and was never open to tourists. Samples originated from limestone walls located immediately below the Apse, in an area of the Diaclase termed the Shaft of the Dead Man.

Within each room, samples were collected from visual marks (when occurring) and unstained surfaces, as previously described (Alonso et al., 2018). Approximately 50 mg of wall material samples (3–6 replicates) were collected using sterile scalpels and placed in liquid nitrogen for transportation to the laboratory, where they were stored at −80°C until DNA extraction. Six samples from Rouffignac, six from Mouflon, five from Reille, and forty‐one from Lascaux (five from Sas‐1, six from Passage B, six from Passage IP, eighteen from Apse, and six from Diaclase) were used for subsequent metabarcoding analyses (Table A1). Sampling was performed in accordance with the cave rules and regulations.

### 2.2 DNA extraction and amplicon sequencing

DNA was extracted using the FastDNA SPIN Kit for Soil (MP Biomedicals) following the manufacturer's instructions, and adapted to the low sample size, as previously described (Alonso et al., 2018). The elution step was achieved using two volumes of 50 μl elution buffer per sample.

The primers 341F (5′‐CCTACGGGNGGCWGCAG‐3′) and 805R (5′‐GACTACHVGGGTATCTAATCC‐3′), which target the V3–V4 region (428 bp fragments), were used for bacterial 16S rRNA gene amplification (Herlemann et al., 2011). The polymerase chain reaction (PCR) cycling protocol consisted of initial denaturation at 95°C for 5 min; followed by 25 cycles of denaturation (95°C for 40 s), annealing (55°C for 30 s), and extension (72°C for 1 min); and a final extension step at 72°C for 7 min. Amplification and sequencing were performed by the Fasteris company using the Illumina MiSeq Reagent Kit with V3 chemistry (600 cycles) in the paired‐end mode, resulting in 2 × 300 bp sequence reads. We used a subset of the sequences obtained by Alonso et al. (2018).

The 441 bp fragments of the 65 kDa heat‐shock protein gene (*hsp*65) were amplified by PCR using the specific primers TB11 (5′‐ACCAACGATGGTGTGTCCAT‐3′) and TB12 (5′‐CTTGTCGAACCGCATACCCT‐3′) from Telenti et al. (1993). Amplifications were performed in a reaction mixture of 50 µl in packaged PCR tubes (PuReTaq Ready‐To‐Go PCR Beads; GE Healthcare; 2.5 U of Taq polymerase PuReTaq, 10 mM Tris–HCl [pH 9], 50 mM KCl, 1.5 mM MgCl_2_, and 200 µM of each deoxynucleoside triphosphate) with primers (0.2–0.4 µM, depending on the template concentration), and DNA (1–40 ng). The PCR cycling protocol consisted of initial denaturation at 94°C for 5 min, then 35 cycles of denaturation (94°C for 60 s), annealing (55°C for 60 s), and elongation (72°C for 60 s; Rodríguez‐Nava et al., 2006). Sequencing, purification, and quality control were performed by the Biofidal Company (http://www.biofidal.com) using the Illumina MiSeq Flow Cell V3 in paired‐end mode, resulting in 2 × 300 bp sequence reads. Overall, 50 and 45 samples were successfully sequenced for the 16S rRNA gene and *hsp*65, respectively, while sequences for both markers were obtained for only 37 samples (Table A1).

### 2.3 Processing and analysis of sequence data

Paired sequence reads obtained from sequencing were merged using Fast Length Adjustment of Short reads (FLASh; Magoč & Salzberg, 2011) with a maximum of 25% mismatches in the overlapping region. The sequences were then filtered and aligned using reference alignments of *hsp*65 gene sequences (this study) or 16S rRNA gene sequences from the Silva database V132 (Quast et al., 2012). Chimeric sequences were removed using the integrated Vsearch tool (Rognes et al., 2016) according to the MiSeq standard operating procedure (MiSeq SOP, February 2018; Kozich et al., 2013) in Mothur v1.39.5 software (Schloss et al., 2009). Sequence libraries were taxonomically assigned in Mothur using the *hsp*65 reference database for *hsp*65 sequences and the recreated SEED database subset of the Silva Small Subunit rRNA Database, release 132 (Yilmaz et al., 2014), adapted for use in Mothur (https://mothur.org/w/images/a/a4/Sylva.seed_v132.tgz), as the reference database for the 16S rRNA gene sequences.

**Figure 2:**
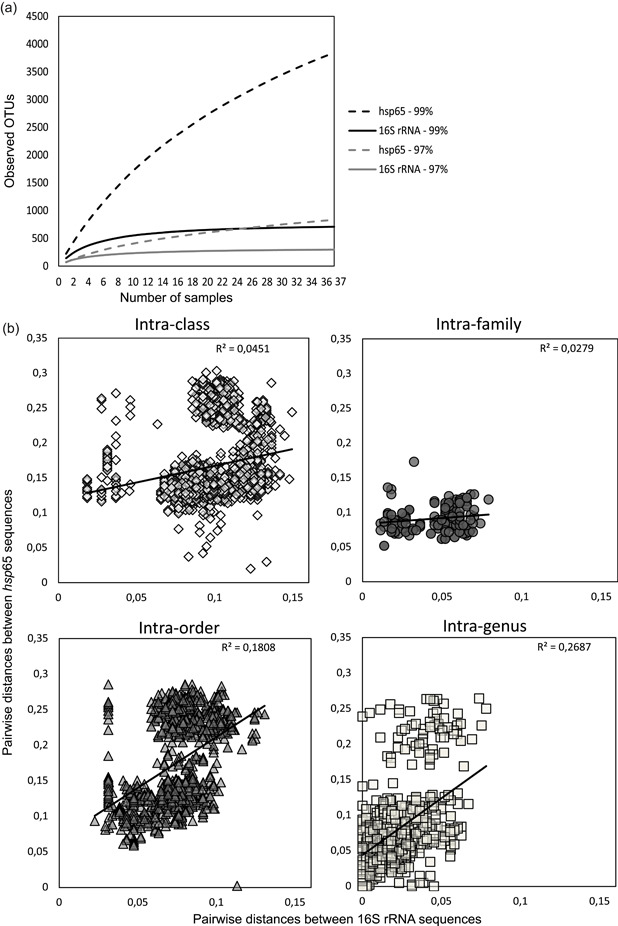
(a) Rarefaction curves for 37 common samples of the *hsp*65 and 16S rRNA genes at the 97% and 99% OTU cutoffs. The *X*‐axis denotes the number of samples, and the *Y*‐axis denotes the number of OTUs. (b) Pairwise molecular distances between sequences of the *hsp*65 (*Y*‐axes) and 16S rRNA gene (*X*‐axes) genes among *Actinobacteria* species from different taxonomical levels with the correlation coefficient (*R* ^2^) for each equation. OTU, operational taxonomic unit

The sequences of plastids and mitochondria, and those not classified in the domain Bacteria (from the 16S rRNA gene sequence library), as well as sequences identified as homologs for *hsp*65 (*groEL*, in the database renamed as GROESL; see the *hsp*65 reference database design section; from the *hsp*65 sequence library), were discarded. The sequence library was clustered into operational taxonomic units (OTUs) using the Uparse pipeline in Usearch v10.0.240 software (OTUs at a 97% cutoff; Edgar, 2013) and Mothur (OTUs at a 99% cutoff). Two cutoffs were applied, as the 97% cutoff is used as a standard threshold for estimating bacterial diversity for the 16S rRNA gene. Therefore, it might underestimate total diversity, especially that of the *hsp*65 marker, which has higher variability than the 16S rRNA gene marker (see Figure 2). The tighter 99% cutoff was used for a more reliable estimation of *Actinobacteria* diversity at the lowest taxonomic ranks, particularly for the *hsp*65 marker (Chen et al., 2013). The OTU table was further processed using tools implemented in Mothur. For 16S rRNA genes, only sequences corresponding to *Actinobacteria* were used.

To compare *Actinobacteria* among the caves, 25 samples for *hsp*65 and 24 samples for 16S rRNA genes were used (Table A1). Apse and Diaclase (using unmarked areas) were selected as representative samples for Lascaux Cave, except for the analysis of the core microbiome (i.e., common OTUs present in all caves), for which all samples from Lascaux Cave samples were included. To examine differences among locations in Lascaux Cave, 33 samples for *hsp*65 (16 from marked areas and 17 from unmarked areas) and 38 samples for the 16S rRNA gene (20 from marked areas and 18 samples from unmarked areas) were used (Table A1). For the 16S rRNA gene marker, samples from the Sas‐1 unmarked area were missing for the analysis, because only a low number of replicates were successfully sequenced. To obtain the highest number of replicates per sample, all samples were included in the analysis, even when they were not common to both markers. A total of 37 common samples for both markers were used to compare these markers, that is, rarefaction analysis, comparison of diversity indices, and several taxa recovered at different taxonomy ranks.

Rarefaction analysis was performed to analyze the richness of the *Actinobacteria* OTUs based on two cutoffs (i.e., 97% and 99%) as a function of sample number. The alpha diversity indices (richness, Chao‐1; evenness, Simpson evenness; and diversity, inverse Simpson) were calculated in Mothur (97% and 99% OTU cutoff). Significant differences among samples were calculated by analysis of variance (ANOVA) with Tukey's post hoc tests (*p* < 0.05; for caves or locations in Lascaux Cave), Student's *t‐*tests, and *F* tests (*p* < 0.05; for marked/unmarked areas) in the Past 4.02 software (Hammer et al., 2001).

Bray–Curtis distance matrices were calculated to describe differences in bacterial community composition among individual samples. *Actinobacteria* communities in the caves, in different locations of Lascaux Cave, and between marked and unmarked areas within each location were compared by nonmetric multidimensional scaling (NMDS) according to both gene markers (97% OTU cutoff). Analysis of molecular variance (AMOVA) and homogeneity of molecular variance (HOMOVA) were calculated in Mothur with 100,000 iterations for the same factors (97% OTU cutoff).

Pairwise comparisons were made with the Metastats method using Fisher's exact tests (White et al., 2009), and the linear discriminant analysis (LDA) effect size (LEfSe; Segata et al., 2011) was calculated to identify the *Actinobacteria* OTUs that differed significantly among the respective caves, locations, and marked/unmarked areas in Lascaux Cave (99% OTU cutoffs). Metastats was also used to compare relative abundances of *Streptomyces* sequences among the marked/unmarked areas (*hsp*65 marker). Venn diagrams were created in Mothur to test the number of OTUs that encompassed the core microbiome for the different caves and rooms (i.e., Sas‐1, Passage, Apse, and Diaclase) in Lascaux Cave (99% OTU cutoff).

Taxonomic classification of OTUs was performed with Mothur using the *hsp*65 and 16S rRNA gene SILVA V132 reference databases, and species‐level identification with the *hsp*65 marker was verified by BLASTN in the online databases of the National Center for Biotechnology Information (NCBI; US National Library of Medicine). In parallel, a phylogenetic analysis based on *hsp*65 was performed using retrieved sequences from caves belonging to most representative OTUs and those from the GenBank database after using the nucleotide BLAST (BLASTN) tool. Retrieved sequences from GenBank were those with the highest similarity with selected OTUs. Multiple alignments were generated using ClustalX (Thompson et al., 2003). MEGA6 software was used to compute the maximum likelihood of neighbor‐joining phylogenetic trees from the observed amino acid sequence divergence (%; Tamura et al., 2013). The taxonomical composition of the core microbiome was constructed using the Krona tool (Ondov et al., 2011). The number of taxa recovered with the common samples by *hsp*65 and 16S rRNA gene analyses at different taxonomic ranks was calculated and displayed using Venn diagrams.

Co‐occurrence networks were used to predict how marked/unmarked areas and different locations influenced the relative abundance of *Actinobacteria* in Lascaux Cave. Spearman correlation coefficients were computed in Mothur for Lascaux Cave OTUs and were categorized based on their significantly different proportions in the marked/unmarked areas, regardless of the location in Lascaux Cave (Metastats) or for the Lascaux Cave locations (LEfSe; 99% OTU cutoff). Only significant (*p* < 0.03) correlations higher than 0.8/0.8 and lower than −0.35/−0.5 for the *hsp*65/16S rRNA gene markers were visualized in Gephi 0.9.2. (M. Bastian et al.) by Fruchterman–Reingold spatialization (Fruchterman & Reingold, 1991). The average degree, eigenvector centrality, and modularity were computed, and the minimum number of links was filtered to five (using a degree range filter).

Figures were plotted using the vegan package (Oksanen et al., 2018) in the R computing environment (R Core Team, 2018) and Inkscape (v0.92; http://www.inkscape.org).

### 2.4 In silico analysis

Differences in the variability of the *hsp*65 and 16S rRNA gene partial sequences between pairs of *Actinobacteria* strains at different taxonomical levels (e.g., intraclass, intraorder, intrafamily, and intragenus) were determined using genomes retrieved from the Integrated Microbial Genomes and Microbiomes database (IMG, University of California, https://img.jgi.doe.gov/). For the analysis, sequences from the available genomes of 47 species from the order Streptomycetales (*Streptomyces, Kitakatospora*, Table A2), and 58 species from the order Corynebacteriales (family Corynebacteriaceae: *Corynebacterium*; family Gordoniaceae: *Gordonia*; family Mycobacteriaceae: *Mycobacterium*; family Nocardiaceae: *Nocardia*, *Rhodococcus*; Table A2) were used. Sequences were aligned and trimmed to the amplified length of both gene markers in BioEdit 7.2.5 software (Hall et al., 2011). Pairwise distances between sequences were calculated in Mothur v. 1.39.5 software (Schloss et al., 2009). Plots were created and the coefficient of determination (*R* ^2^) was calculated in Microsoft Excel (2016).

### 2.5 *hsp*65 reference database design

For taxonomic assignment using the *hsp*65 marker, the reference database of the *Actinobacteria hsp*65 gene was constructed similarly to that of the *rpoB* marker by (Ogier et al., 2019). Here, we built a new reference library based on the *hsp*65 gene encompassing both newly obtained and public sequences from the most important *Actinobacteria* taxa. Part of this new reference library was constructed with *Nocardia* and *Gordonia* sequences from all type strains, from strains from clinical collections (supplied by the French Observatory for Nocardiosis, OFN http://ofn.univ‐lyon1.fr), and from environmental strains (supplied by CRB‐EML http://eml‐brc.org/) sequenced by our means for which sequence quality was verified by double sense sequencing. For the *Mycobacterium* genus, *hsp*65 sequences were available via the BIBI database (https://umr5558-bibiserv.univ-lyon1.fr/lebibi/lebibi.cgi), which is fairly robust as it contains all described *Mycobacterium* species. The rest of the sequences was collected from the online IMG and NCBI databases using sequences homologous to the 65 kDa heat‐shock protein (*hsp*65) of the reference strain *Nocardia asteroides* ATCC 14759 (Rodríguez‐Nava et al., 2006) amplified by the TB11 and TB12 primers (Telenti et al., 1993). Paralogs of heat‐shock proteins, such as *groEL* genes from *Escherichia coli* (Colaco & MacDougall, 2014; Duchêne et al., 1994; C. M. S. Kumar et al., 2015), were identified using maximum likelihood, FastTree 2.1 (Price et al., 2010), and were retained in the reference database (renamed GROESL sequences) as an outgroup that enabled amplified sequences belonging to this variant to be discarded. This database was named “ACTIhsp65_V1.0.0.fas” and is available at https://doi.org/10.5281/zenodo.5576073

The *hsp*65 database contained 198 genera and 1066 unique taxa, the whole represented by 5165 sequences, of which 1782 were denoted as GROESL at the time of the analysis. TB11 and TB12 primer complementarity with sequences from the reference database was evaluated using the OligoAnalyzer 3.1 tool (http://www.idtdna.com/calc/analyzer). Percent coverage of primers for *Actinobacteria* genomes from different classes and orders was determined with primer BLAST using the RefSeq reference genomes (NCBI). Only targets with no mismatches for the last three nucleotides at the 3′ end and no more than four overall mismatches were included.

## 3 RESULTS

### 3.1 Sequence polymorphism of *hsp*65 versus 16S rRNA genes for cave *Actinobacteria*

The rarefaction curves of 16S rRNA genes for both 97% and 99% cutoffs were more tilted, indicating an accumulation of identical OTUs due to their repeated sampling, which did not increase with a stricter cutoff, in contrast to *hsp*65 (Figure 2a). These results are consistent with the in silico analysis of *hsp*65 and 16S rRNA gene partial sequences obtained from 105 genomes (47 species from the order Streptomycetales and 58 species from the order Corynebacteriales) available on IMG. Pairwise distances between *hsp*65 sequences, even for closely related species, were higher than those between 16S rRNA genes (Figure 2b).

**Figure A1:**
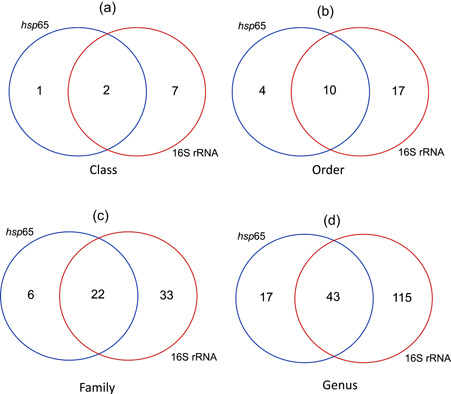
Venn diagrams showing the number of different *Actinobacteria* taxa recovered with *hsp*65 (blue) or 16S rRNA gene (red) at different taxonomic ranks. (a) Class, (b) order, (c) family, and (e) genus

The *hsp*65 primers targeted *hsp*65 sequences from the reference database with lower numbers of mismatches compared with the outgroup (i.e., distant homologs renamed in the database as GROESL, *GroEL* of *E. coli*, and Chloroflexi members; Table A3). However, the primers only targeted select groups from the class *Actinobacteria*, as demonstrated in the analysis with representative genomes from online databases of the NCBI (US National Library of Medicine; Table A4). The number of taxa identified at class or lower taxonomic levels is shown in Figure A1. Data indicated that 20% of the classes, 32% of the orders, 36% of the families, and 25% of the genus‐level taxa detected with *hsp*65 were also recovered through 16S rRNA gene analysis. Although there was a trend for a higher number of taxa uniquely detected by the *hsp*65 marker at lower taxonomic ranks, contrary to the 16S rRNA gene marker, *hsp*65 sequences detected a lower number of taxa overall. However, at the species level, s 168 species were recovered with the *hsp*65 marker only, and the highest species numbers were found within the *Streptomyces*, *Mycobacterium*, and *Nocardia* genera (Table A5).

In summary, the 16S rRNA gene marker was more suited for broad taxonomic profiling of the *Actinobacteria* community. In comparison, the *hsp*65 marker could help distinguish between selected *Actinobacteria* species, which the 16S rRNA gene marker failed to achieve; therefore, it complemented the results obtained with the 16S rRNA gene marker (Table A5).

### 3.2 *Actinobacteria* diversity in caves based on *hsp*65 versus 16S rRNA genes

**Figure A2:**
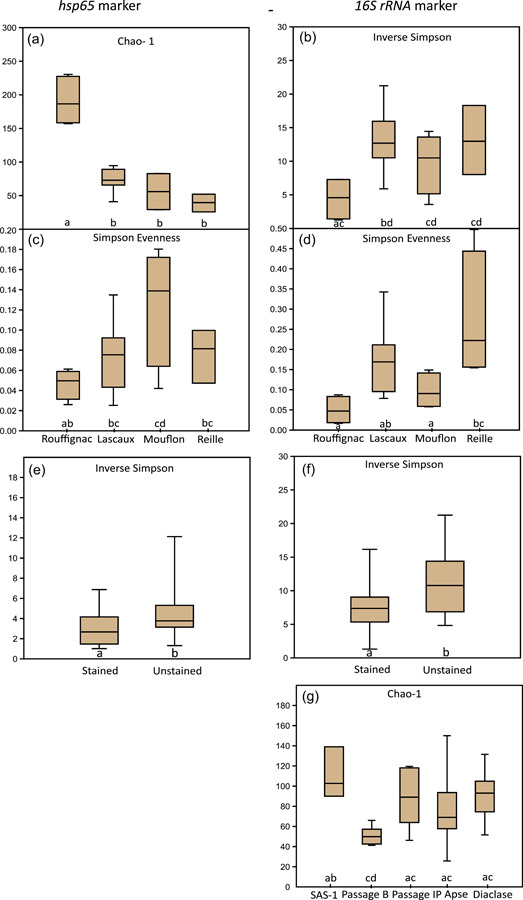
Indices of alpha diversity (richness, Chao‐1; evenness, Simpson evenness; diversity, inverse Simpson) for *Actinobacteria* communities among caves, different locations within Lascaux Cave, and marked/unmarked areas. Significant differences between groups are shown with letters (ANOVA and Tukey post hoc test; *p* < 0.05; 97% OTU cutoff). ANOVA, analysis of variance; OTU, operational taxonomic unit

In the diversity analysis based on 37 common samples of both markers, the *hsp*65 marker recorded higher richness (Chao‐1 index) and evenness (Simpson evenness index) than the 16S rRNA gene marker when considering the 99% OTU cutoff (Table A6). The diversity analysis based on all samples indicated that Rouffignac Cave exhibited the highest *Actinobacteria* richness (Chao‐1 index, for the *hsp*65 marker; Figure A2) but lower evenness (Simpson evenness index; for both markers; Figure A2) and diversity (inverse Simpson index; for the 16S rRNA gene marker; Figure A2) among other caves. In contrast, the Lascaux and Rouffignac Caves had lower evenness than the pristine Reille (for the 16S rRNA gene marker) and Mouflon (for the *hsp*65 marker; Figure A2) caves. When comparing locations within Lascaux Cave, significantly higher *Actinobacteria* richness was detected in Sas‐1 compared with Passage B using only the 16S rRNA gene marker (Figure A2). The average diversity of the unmarked areas was higher than that of the marked areas for both markers (Figure A2).

### 3.3 *Actinobacteria* community structure in caves based on *hsp*65 versus 16S rRNA genes

**Figure 3:**
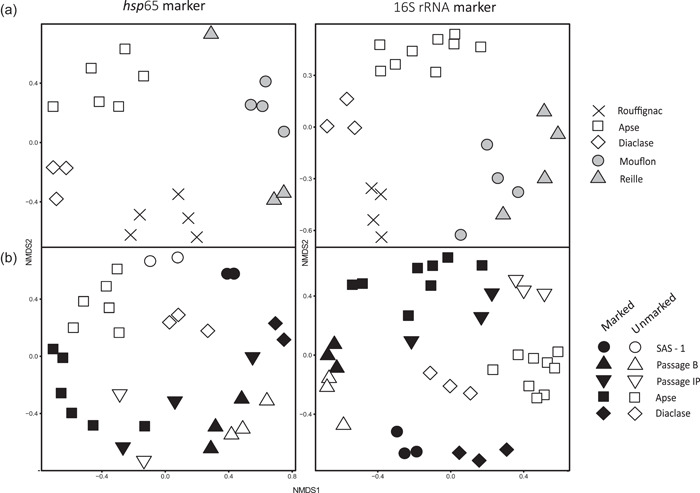
Sammon projection of nonmetric multidimensional scaling (NMDS) based on the Bray–Curtis distance matrices of the *hsp*65 and 16S rRNA gene markers for different caves (a) and Lascaux Cave locations, and marked/unmarked areas (b). The *F* and *p* values of overall AMOVA (97% OTU cutoff) are indicated in Table A7. AMOVA, analysis of molecular variance; OTU, operational taxonomic unit

NMDS analysis was conducted to compare the *Actinobacteria* communities in caves (97% OTU cutoff). These communities did not differ significantly between pristine caves, Reille and Mouflon (AMOVA), based on both *hsp*65 and 16S rRNA genes. NMDS distinguished three groups of communities corresponding to Lascaux, Rouffignac, and the two pristine caves, Reille and Mouflon, which was further supported by AMOVA (Table A7 A). The AMOVA comparison of anthropized (Lascaux and Rouffignac) versus pristine (Reille and Mouflon) caves revealed that they significantly differed according to both markers (*hsp*65: *F* = 3.986, *p* < 0.001; 16S rRNA genes: *F* = 8.610, *p* < 0.001). Within Lascaux Cave, the *Actinobacteria* communities differed largely according to location (i.e., Sas‐1, Passage B, Passage IP, Apse, and Diaclase) with both markers, except that differences between Passage IP, Sas‐1 (both markers), and Diaclase (*hsp*65) were not significant due to the low number of samples (Table A7 A). Overall, the effect of surface alterations (i.e., marked vs. unmarked areas) in Lascaux Cave was significant for both markers (Table A7 A), with higher *Actinobacteria* homogeneity in unmarked versus marked areas (Table A7 B). Passage B presented the highest community stability compared with Passage IP, Apse, and Diaclase (Table A7 B), which was reflected in the NMDS plot, where the marked and unmarked areas in Passage IP were the least separated (Figure 3).

### 3.4 Core and cave‐specific *Actinobacteria* microbiomes based on *hsp*65 versus 16S rRNA genes

**Figure A3:**
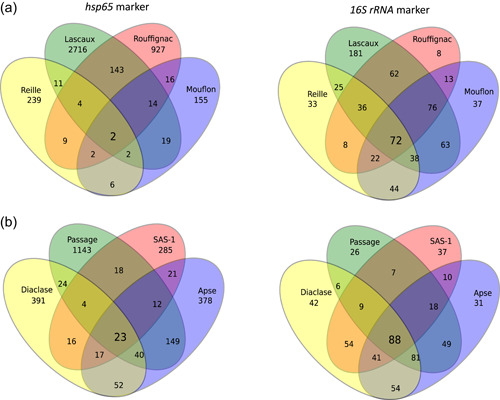
Venn diagrams showing unique and shared OTUs between (a) different caves and (b) different locations within Lascaux Cave based on *hsp65* and 16S rRNA gene markers (99% OTU cutoff). OTU, operational taxonomic unit

**Figure A4:**
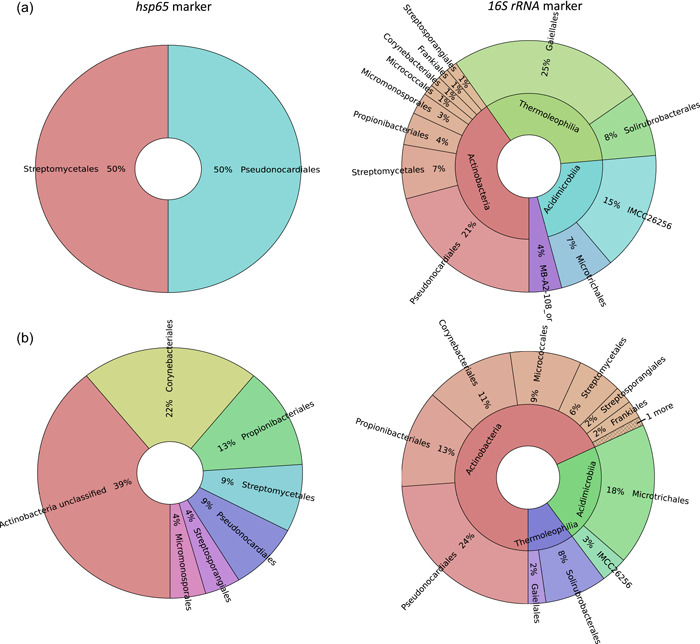
Taxonomic compositions of the core microbiome between (a) caves and (b) different locations within Lascaux Cave based on the *hsp*65 and 16S rRNA gene markers (99% OTU cutoff). OTU, operational taxonomic unit

Venn diagrams showed that the number of OTUs shared between different caves (Rouffignac, Lascaux, Mouflon, and Reille) was two (0.04%) for the *hsp*65 marker and 72 (11%) for the 16S rRNA gene marker (Figure A3). Similarly, the number of OTUs shared between different Lascaux rooms (Sas‐1, Passage, Apse, and Diaclase) was 23 (0.89%) for the *hsp*65 marker and 88 (15.9%) for the 16S rRNA gene marker. The core microbiome was mainly constituted by Pseudonocardiales and Streptomycetales, as determined by *hsp*65, and Gaiellales, Pseudonocardiales, and strain IMCC26256_ge, as determined by 16S rRNA genes (Figure A4).

**Figure 4:**
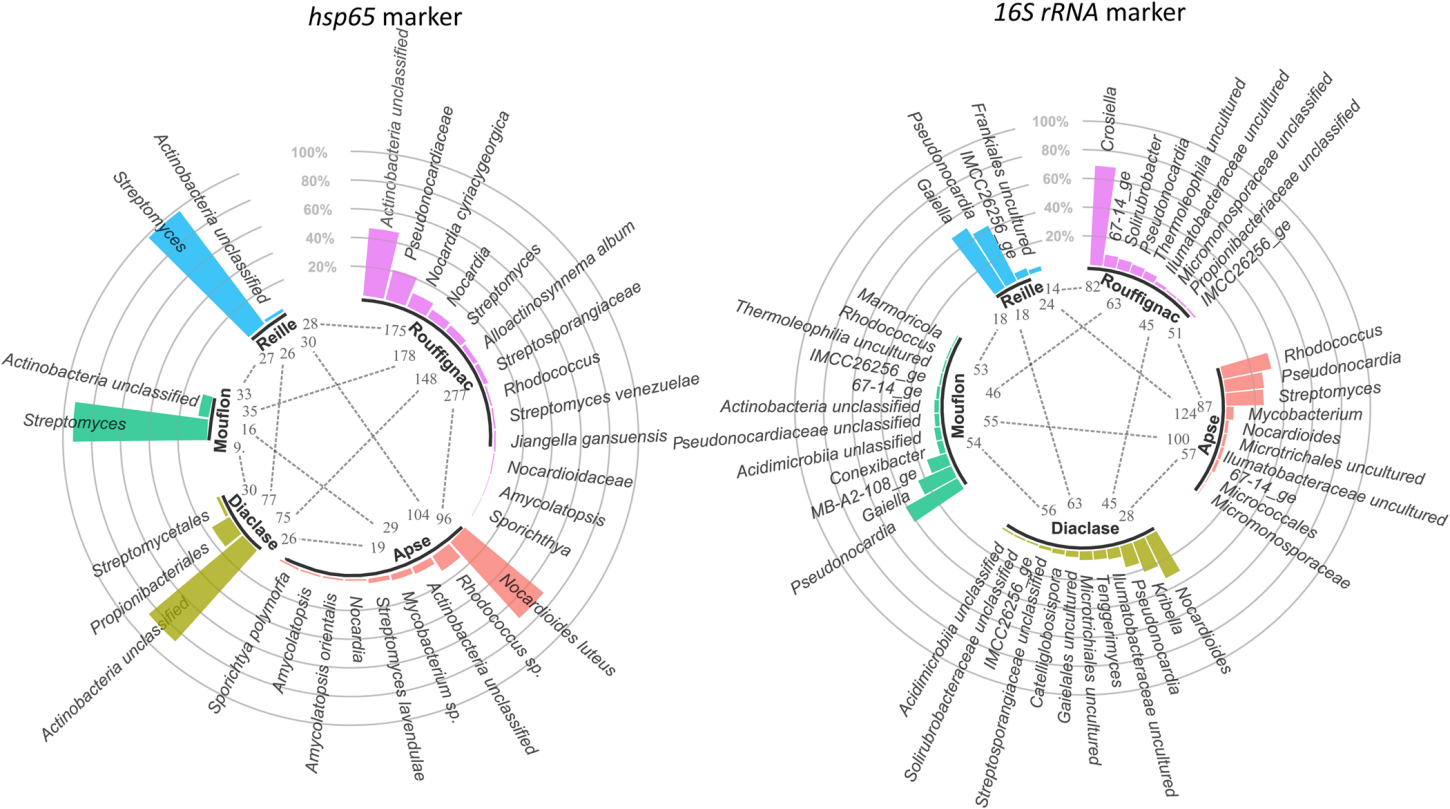
Significantly different OTUs between pairs of caves (Metastats, *p* < 0.005). For each cave, the number of OTUs that differed proportionally from other caves and taxonomically assigned OTUs proportionally the most abundant in the respective cave is indicated (99% OTU cutoff). OTU, operational taxonomic unit

Pairwise comparison of cave conditions (Rouffignac, Lascaux's Apse, and Diaclase, Mouflon, Reille) using significantly different OTUs (Metastats; White et al., 2009) showed that Rouffignac (based on *hsp*65) and Lascaux's Apse (based on 16S rRNA genes) were most enriched in OTUs, separating them from the other caves (Figure 4). Among them, Pseudonocardiaceae, *Crosiella*, and *Nocardia* were the most proportionally abundant taxa that distinguished Rouffignac (for both markers), while *Nocardioides*, *Rhodococcus*, and *Pseudonocardia* distinguished Lascaux's Apse from the other caves and Diaclase. *Mycobacterium* was uniquely found in Lascaux's Apse based on both markers. Pristine caves were typified by *Streptomyces* (*hsp*65 marker), Gaiellales, and Pseudonocardiales, and the environmental clones MB‐A2‐108_ge and IMCC26256_ge (16S rRNA genes; Figure 4).

### 3.5 Proportions of cave *Actinobacteria* in sequence libraries based on *hsp*65 versus 16S rRNA genes

A total of 1,799,680 *hsp*65 and 1,919,236 16S rRNA gene sequences were obtained, out of which 696,716 *hsp*65 gene sequences were mapped to 968 (97% cutoff) and 4269 (99% cutoff) OTUs, while 136,183 16S rRNA gene sequences were mapped to 299 (97% cutoff) and 718 (99% cutoff) OTUs.

Within that, 696,007 *hsp*65 and 99,980 16S rRNA gene sequences were allocated to the *Actinobacteria* class, whereas 293,923 and 3803, respectively, could not be allocated to a defined order. Concerning the *hsp*65 gene, the *Streptomyces* genus was the most proportionally abundant, with 69,245 sequences representing about 9.9% of the identified *Actinobacteria*; however, 9283 sequences from this genus could not be affiliated to a specific species within the *Streptomyces* genus. The *Mycobacterium* and *Nocardia* genera were also important groups representing about 7.8% and 1.3% of the identified *Actinobacteria*, respectively.

For the 16S rRNA gene data, the *Streptomyces* genus was the second most represented after *Pseudonocardia*, with 13,263 sequences representing about 9.7% of the identified *Actinobacteria*. The *Mycobacterium* and *Nocardia* genera were other important groups representing about 0.79% and 0.85% of the identified *Actinobacteria*, respectively.

**Figure 5:**
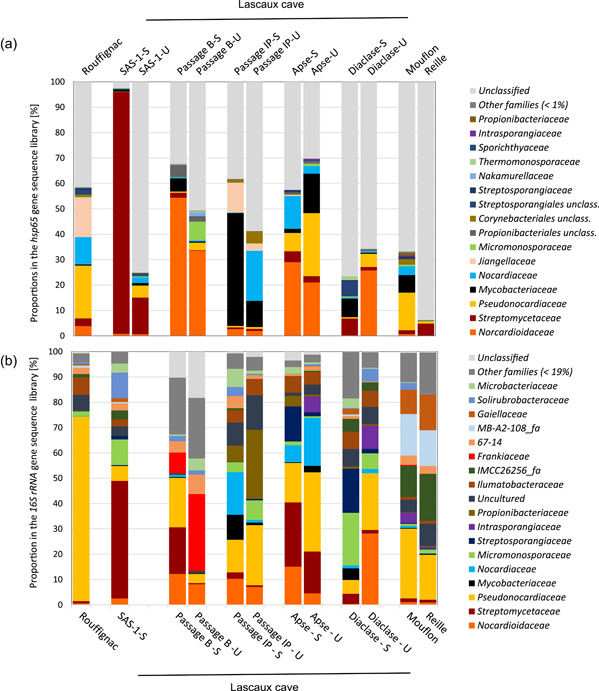
Average proportions of *Actinobacteria* families based on *hsp*65 (a) and 16S rRNA gene (b) sequence libraries from different caves (Rouffignac, Lascaux, Mouflon, and Reille), Lascaux Cave locations (Sas‐1, Passage B, Passage IP, Apse, and Diaclase) and marked (S)/unmarked (U) areas within Lascaux

Taxonomic analysis showed that 27 families were identified by both gene markers, but five families were uniquely identified by the *hsp*65 marker but not by the 16S rRNA gene marker, while 40 families were identified by the 16S rRNA gene marker but not by the *hsp*65 marker. Families uniquely found by the *hsp*65 marker were *Jiangellaceae*, Actinobacteria incertae sedis, *Brevibacteriaceae*, *Nocardiopsaceae*, and *Gordoniaceae* (Figure 5).

**Figure A5:**
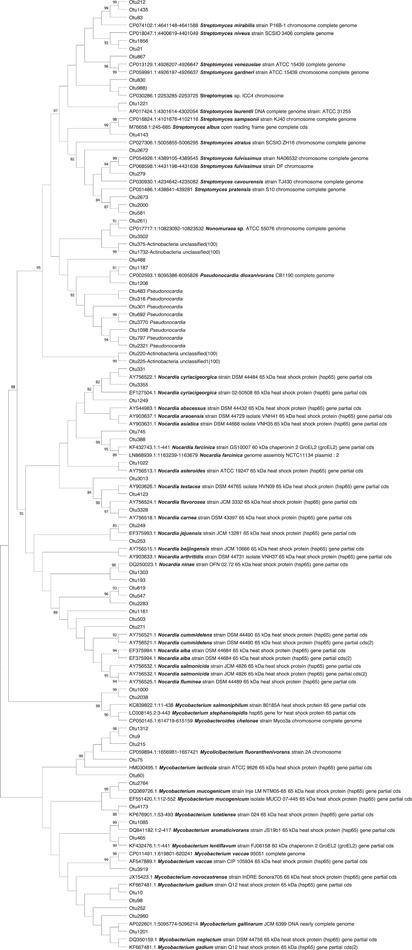
Phylogenetic tree showing the taxonomic diversity and allocation of *hsp*65 sequences from caves retrieved by a metabarcoding approach. OTUs were used to build this figure. The evolutionary history was inferred using the neighbor‐joining method (Saitou & Nei, 1987) and reveals the relationship of sequences from caves obtained in this study with sequences belonging to type and reference strains classified within the *Actinobacteria* class. The tree is based on the Kimura two‐parameter method (Kimura, 1980) with the confidence values of the branches determined by bootstrap analyses (Felsenstein, 1985) based on 1000 replicates. Only values >80% are shown at the nodes. This analysis involved 116 nucleotide sequences. The scale bar represents 0.020 substitutions per nucleotide position. Evolutionary analyses were conducted in MEGA 6 (S. Kumar et al., 2018). OTU, operational taxonomic unit

**Figure A6:**
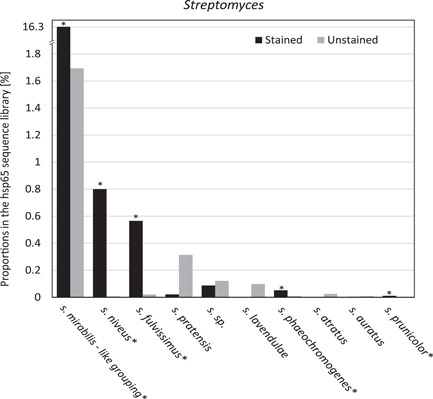
Average proportions of species within the genus *Streptomyces* in the *Actinobacteria hsp*65 amplicon sequence libraries significantly different in marked (S) and unmarked areas (U) of Lascaux Cave (Metastats, *p* < 0.05).

**Figure A7:**
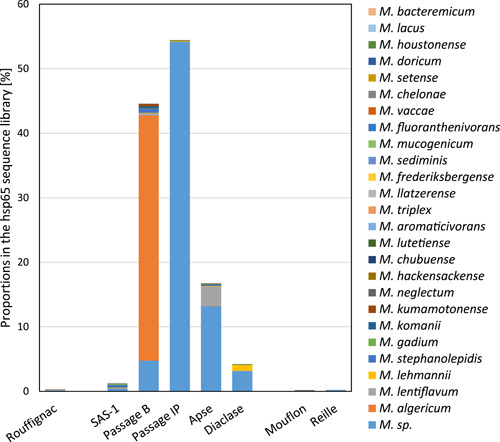
Average proportions of genus *Mycobacterium* in the *Actinobacteria hsp*65 gene amplicon sequence libraries in different caves (Rouffignac, Lascaux, Mouflon, and Reille) and different locations within Lascaux Cave (Sas‐1, Passage B, Passage IP, Apse, and Diaclase).

**Figure A8:**
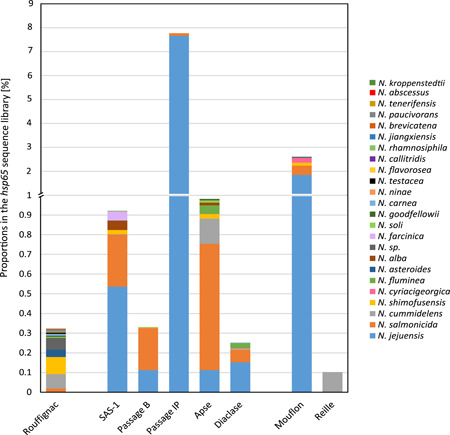
Average proportions of genus *Nocardia* in the *Actinobacteria hsp*65 gene amplicon sequence libraries in different caves (Rouffignac, Lascaux, Mouflon, and Reille) and different locations within Lascaux Cave (Sas‐1, Passage B, Passage IP, Apse, and Diaclase).

*Nocardioidaceae*, *Streptomycetaceae*, and *Pseudonocardiaceae* dominated the sequence libraries of both gene markers when considering all cave conditions together. On the basis of both gene markers, there was a higher proportion of *Streptomycetaceae* in marked areas compared with unmarked areas of Lascaux Cave (Figure 5). With a closer focus on *Streptomyces* using only the *hsp*65 marker, which can identify species or species‐like groups, an *S. mirabilis*‐like grouping (no more accurate association could be made and this was supported by a bootstrap of just over 60%, Figure A5), *S. niveus* and *S. fulvissimus* (bootstrap > 80%) dominated in marked areas within Lascaux Cave (Metastats *p* < 0.005; Figure A6). This marker could also identify other species, such as *S. exfoliatus* and *S. albus*, with good discriminatory power (supported by a high phylogenetic resolution *n* = 99% bootstrap) in Rouffignac. The opposite result was found for *Pseudonocardiaceae*, which were present in higher proportions in unmarked versus marked areas in Lascaux Cave, except for Passage IP (by *hsp*65) and Passage B (by 16S rRNA gene marker; Figure 5). Among the most proportionally abundant obtained OTU sequences belonging to *Pseudonocardiaceae*, none could be associated with a precise species. *Mycobacteriaceae* were found to be highly represented in Lascaux Cave, and the *hsp*65 marker also identified them in Mouflon. Three of the most proportionally abundant *Mycobacteria* species in Lascaux Cave uncovered with the *hsp*65 marker were *M*. sp., *M. algericum*, mainly in Passage B, and *M. lentiflavum* (Figure A7). *Jiangellaceae* was a typical group in Rouffignac and Passage IP (*hsp*65 marker). Closer analysis of *Nocardiaceae* with the *hsp*65 marker uncovered the greatest richness of *Nocardia* species in Rouffignac (including *N. carnea, N. paucivorans*, and *N. abscessus*). Moreover, high proportions of *N. jejuensis* were found in Lascaux and Mouflon compared with *N. cummidelens* in Rouffignac, Apse, and Reille (Figure A8). *N. cyriacigeorgica* was only found in Mouflon Cave and *N. farcinica* only in SAS‐1. Both pristine caves presented similar compositions of *Actinobacteria* communities, especially *Gaiellaceae*, taxa related to the environmental clones MB‐A2‐108_fa and IMCC26256_fa (16S rRNA gene marker), and high proportions of unclassified *Actinobacteria* (*hsp*65). High proportions of unclassified *Actinobacteria* were found in Lascaux's Diaclase, as found in the pristine caves (Figure 5).

The phylogenetic tree encompassing sequences of the most representative OTUs (99% cutoff) obtained in this study, together with their corresponding closest sequences obtained from GenBank is shown in Figure A5. This analysis revealed that the most proportionally abundant *Mycobacterium* (*M. algericum* and *M. lentiflavum*), *Streptomyces* (*S. mirabilis*‐like grouping, *S. niveus* and *S. fulvissimus*), *Nocardia* (*N. jejuensis*, *N. salmonicida/cummidelens* clade, *N. cyriacigeorgica*, and *N. farcinica*), and *Pseudonocardia* species are supported by a bootstrap >80%. Less represented species were also identified; for example, *M. fluoranthenivorans, M. mucogenicum*, and *M. stephanolepidis/salmoniphilum* clade, *M. gallinarum* (bootstrap > 80%), *S. pratensis*, and *S. albus/sampsonii* clade (bootstrap > 90%); and *N. ninae* (bootstrap = 98%). Identification of these OTUs according to the *hsp*65 reference database and corresponding closest sequences obtained from GenBank are listed in the Supplementary Table at https://doi.org/10.5281/zenodo.6312516.

### 3.6 Co‐occurrence of *Actinobacteria* in Lascaux Cave based on *hsp*65 versus 16S rRNA genes

**Figure 6:**
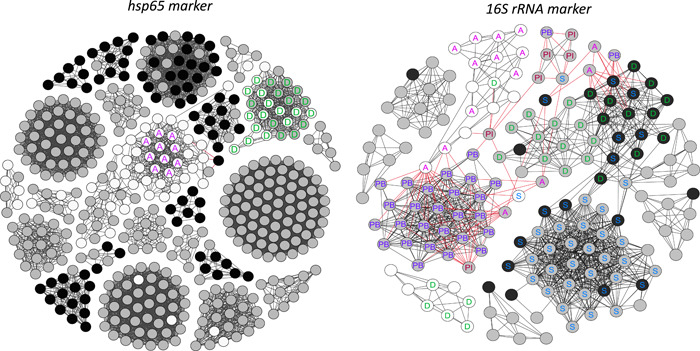
Co‐occurrence networks of *Actinobacteria* OTUs: (a) *hsp*65 marker and (b) 16S rRNA gene marker; OTUs differed significantly between marked (black) and unmarked (white) areas in Lascaux Cave, and those that did not differ between these areas (gray) using Metastats (*p* < 0.05). The letters indicate OTUs that were specific for the respective Lascaux Cave locations (S, Sas‐1; PB, Passage banks; PI, Passage inclined planes; A, Apse; and D, Diaclase) using LEfSe (*p* < 0.03). Strong significant connections (Spearman's correlation >0.8 and <0.5 for 16S rRNA gene and <0.35 for *hsp*65) are displayed (99% OTU cutoff). Red lines indicate negative correlations and black lines indicate positive correlations. OTU, operational taxonomic unit

Co‐occurrence networks revealed Spearman correlations between the relative abundances of *Actinobacteria* OTUs in Lascaux Cave (Figure 6), for both Lascaux locations (calculated by LEfSe; Segata et al., 2011) or marked/unmarked areas (calculated by Metastats; White et al., 2009). More co‐occurrence connections were identified for *hsp*65 than for the 16S rRNA gene. For the *hsp*65 marker, no more correlated OTUs were indicated by LEfSe or Metastats, as typical for the Lascaux locations or marked/unmarked areas, respectively. Negative correlations between the OTUs were only identified with the 16S rRNA gene marker.

Clusters of correlated OTUs dominated the unmarked areas, which also differed significantly in the Apse and Diaclase based on both gene markers (Figure 6). Additionally, OTU clusters that dominated the marked areas were typical for the Diaclase and Sas‐1 areas based on the 16S rRNA gene marker. There was a correlation between OTUs from such distinct areas (Diaclase and Sas‐1), suggesting that the marked‐area factors affected the *Actinobacteria* communities in both locations (Figure 6). However, the *hsp*65 gene marker revealed that the OTU clusters typical of marked areas were not typical of any Lascaux Cave locations. Finally, based on the 16S rRNA gene marker, the highest number of correlations between OTUs that did not differ between the marked and unmarked areas was found for those that dominated in Passage B, Sas‐1, and Diaclase (Figure 6). For those OTUs, the location factor was more influential than the marked/unmarked areas. Networks based on both markers included variable actors (except for the OTUs in unmarked areas of Apse and Diaclase, regardless of marked/unmarked areas), which indicated that each marker covered different fractions of the community and their interactions.

## 4 DISCUSSION

This study represents an attempt to exploit the potential of *hsp*65 as a taxonomic marker to assess the whole *Actinobacteria* community in an extreme environment, such as caves, highlighting its advantages over the 16S rRNA gene marker. When comparing both genes, Venn results revealed higher variability for *hsp*65 sequences compared with *Actinobacteria* 16S rRNA gene sequences, and higher OTU richness (at the 99% cutoff) for *Actinobacteria*. Moreover, using the *hsp*65 marker, we found 168 species, which could not be identified in the same samples using the 16S rRNA gene marker. Therefore, the low interspecific polymorphism of 16S rRNA genes leads to an underestimation of diversity, which might be compensated for by using the *hsp*65 marker, as found for other protein‐coding genes, such as *rpoB* (Vos et al., 2012). However, a higher number of higher‐rank taxa were identified with the 16S rRNA gene compared with the *hsp*65 marker, which highlights gaps in the *hsp*65 reference database, preventing the complete taxonomic assignment of sequences. Another reason might be related to primer mismatches. Indeed, in silico analysis revealed that the primers amplified the gene from diverse *Actinobacteria* genomes; however, targets without perfect homology might be amplified with lower efficiency, leading to the underestimation of some taxa (Deagle et al., 2014). Overall, the data indicated that *hsp*65 is a suitable complement for the 16S rRNA gene marker in high‐throughput sequencing methods for *Actinobacteria*, especially for taxonomic analysis at the species level; however, primer bias may need to be considered for community analyses at higher taxonomic ranks. Moreover, the *hsp*65 reference database is biased against representation in nature, due to uneven selection of restricted taxa, especially compared with those available in online databases. The 16S rRNA gene is available to the scientific community for all species because it must be mandatorily supplied as part of the submission. However, *hsp*65 gene submission is not necessary when describing a new species, explaining the lack of databases. The availability of bacterial genomes is increasing and in the future, many more sequences will be added that will increase the coverage of our database, allowing for more complete detection of taxa within the *Actinobacteria*.

In this study, the combination of *hsp*65 and 16S rRNA gene markers revealed that *Actinobacteria* diversity could vary according to anthropization in caves, on both higher (i.e., between caves) and lower (i.e., between locations within one cave) spatial scales. Indeed, the actinobacterial communities of anthropized (Lascaux and Rouffignac) and pristine caves (Reille and Mouflon) differed. The occurrence of these caves in the same region and limestone vein probably facilitated this observation, as geographic distance (Barraclough et al., 2012) and geological type (Zhu et al., 2019) are significant factors influencing cave communities. The number of *Actinobacteria* that could be identified as part of the core microbiome was relatively low, which suggested that different cave features can lead to different microbial communities (Alonso et al., 2019). Nevertheless, taxa belonging to this core microbiome (Pseudonocardiales, Streptomycetales, and Gaiellales) can resist anthropogenic disturbance (Shade et al., 2012).

Mouflon and Reille are pristine caves with evenly distributed taxa in the *Actinobacteria* community, possibly as a result of their stable environments without man‐made disturbances (Mammola, 2019). Typical pristine cave taxa that were identified included Gaiellales (Zhu et al., 2019) and *Actinobacteria* related to the environmental clone MB‐A2‐108_ge (De, 2019; B. Zhang et al., 2019), according to the 16S rRNA gene marker. High proportions of *Streptomyces* and unclassified *Actinobacteria* based on *hsp*65 data suggest that each marker identifies particular *Actinobacteria* community members. Less anthropized areas, such as the pristine caves of Reille and Mouflon, and the Diaclase area of Lascaux, yielded a higher proportion of “*Actinobacteria* unclassified” when using the *hsp*65 gene compared with the 16S rRNA gene in the same zones. Thus, these areas host species of families whose *hsp*65 gene sequences are missing in our database. Species may already be described for which the *hsp*65 sequence is not available, or they may simply represent new and as yet undescribed species. Therefore, our database, which was conceived to reach the species level, seems to be more suitable to identify sequences derived from anthropized environments.

Environmental selection in oligotrophic habitats may promote rapid molecular evolution (Kuo & Ochman, 2009; Sagova‐Mareckova et al., 2015) and result in a high number of novel species isolated from caves (De, 2019). A similar result was also found for the *Streptomyces* genus (Hamm et al., 2017), whose genomes may undergo a high rate of evolution (Cheng et al., 2015). For pristine caves, high proportions of *Streptomyces* were obtained from the Diaclase, which is an isolated compartment that has been exposed to little human impact within the anthropized Lascaux Cave. This observation seemed to confirm the hypothesis of Rangseekaew et al., who reported that, not only individual caves but also less‐exposed locations within anthropized caves maintained typical *Actinobacteria* cave communities, which probably include novel taxa (Rangseekaew & Pathom‐Aree, 2019).

Contrary to a previous study based on the whole bacterial community (Alonso et al., 2019), high richness but low evenness was observed in the visited Rouffignac Cave compared with the pristine caves, based on *hsp*65 analysis. The high richness of *Actinobacteria* in this cave might reflect a disturbance effect, which promoted the cohabitation of ecologically different microorganisms (Galand et al., 2016).

In all four caves, the most abundant *Nocardia* species were nonpathogenic, typically associated with caves. In Lascaux and Mouflon *N. jejuensis* dominated, a species that was first isolated from a natural cave, while in Reille and Rouffignac *N. cummidelens* dominated, a species previously isolated from rocks of visited Altamira cave (Jurado, Fernandez‐Cortes, et al., 2009). Conversely, certain opportunistic pathogenic *Nocardia* species were also identified. In Rouffignac, *N. carnea* (Boiron et al., 1992), *N. paucivorans* (Watanabe et al., 2006), *N. testacea* (Taj‐Aldeen et al., 2013), and *N. abscessus* complex (Kageyama et al., 2004) were identified, this latter already found in anthropized environments (Vautrin et al., 2021). Other opportunistic pathogenic species such as *N. farcinica* were found in SAS‐1, which is one of the most anthropized areas of Lascaux Cave. Its presence may be associated with human disturbance. The OTUs associated with this species were very close to the GenBank sequences obtained from a patient with pulmonary coinfection with *M. tuberculosis* (KF432743.1) and Madura foot (CP031418.1; Y. Y. Zhang et al., 2013). Other opportunistic pathogenic species of *Streptomyces* genera such as *S. albus* could also be found, at a low proportion, in Rouffignac cave, which may also be a consequence of human presence (Martín et al., 2004).

However, pathogenic species were also found in pristine caves, including *N. cyriacigeorgica*, which is one of the most frequent *Nocardia* opportunistic pathogenic species in France. The finding in Mouflon represents the first detection of this species in caves. Thus, this species may belong to the autochthonous core microbiome of the caves. The OTUs associated with this species were close to the GenBank sequences obtained from patients (EF127505.1, EF127507.1, and EF127504.1) and urban sediments (VBUT00000000.1) positioned in phylogroups I and III of the *N. cyriacigeorgica* complex (Schlaberg et al., 2008; Vautrin et al., 2021). Our study has succeeded in positioning retrieved sequences in different phylogroups inside this complex.

As shown by Zoropogui et al. (2013), the whole genome of *N. cyriacigeorgica* presents the acquisition of new genetic elements allowing it to thrive in multiple environments. This is confirmed by its isolation in a cave (this study), in the urban sediments of an infiltration basin (Vautrin et al., 2021), or even in humans. Its presence under different extreme environments could explain the evolution of this species into several different phylogroups, which may possess different levels of virulence. The sequences found in this cave may belong to the less virulent and most preserved phylogroups, unlike phylogroup II (the most virulent of this species), which was not identified here.

Other opportunistic pathogens belonging to the Mycobacteriaceae family were also identified in Lascaux Cave. The two most proportionally abundant species, *M. lentiflavum* and *M. algericum*, are causal agents of pulmonary infections in humans (Chida et al., 2021) and animals (Sahraoui et al., 2011), respectively. In addition, the presence of these species has been reported in aquatic mediums (Makovcova et al., 2015; Moradi et al., 2019; Mrlik et al., 2012). OTUs obtained in our study that belonged to these two species were close to the GenBank sequences; one of them was obtained from *Pinna nobilis* (GenBank submission code MN854410.1). Thus, the presence of these species could be due to human input or the presence of water, possibly due to infiltration.

This study reports the occurrence of the most frequent opportunistic pathogenic *Actinobacteria* in France from caves. It is unknown whether environmental strains from these species present any pathogenic potential; however, the occurrence of these species in caves suggests a possible allochthonous input by tourists. In turn, this raises potential health concerns, as previously proposed (Jurado et al., 2010), although *Actinobacteria* disease related to cave visitation has never been reported. In contrast, Jiangellaceae are a family typical of pristine caves (Rangseekaew & Pathom‐Aree, 2019) and are another important group in Rouffignac Cave. According to the intermediate disturbance hypothesis (Roxburgh et al., 2004), the coexistence of taxa typical of pristine or anthropized conditions in the same habitat suggests that Rouffignac Cave is under intermediate environmental pressure.

The actinobacterial communities in caves were highly specific even within very short distances (Zhu et al., 2019) since each Lascaux room hosted its own *Actinobacteria* population. Lascaux Cave was typified by high relative abundances of *Mycobacterium*. Similar to *Nocardia* in Rouffignac, this group can signify external contamination (Jurado et al., 2010; Modra et al., 2017), although it is commonly found in natural environments (Kopecky et al., 2011). Confirming the previous results from Alonso et al. (2018), which were based on whole bacterial community analysis, the different geological substrates of Lascaux's Passage had a stronger role than the occurrence of visual marks; however, the marks had formed in the past and were stable. The effect of the geological substrate could also be observed when comparing Diaclase and Apse, because these two locations displayed different *Actinobacteria* communities, especially in unmarked areas, as shown by the network analysis. Relative isolation and distance from the cave entrance may represent efficient filters for alien microorganisms and enable the maintenance of the cave oligotrophic community, typical for unmarked areas (Cuezva et al., 2009; Mammola, 2019).

Our results showed that the *Actinobacteria* community, mainly *Streptomyces* taxa, varied according to the presence of visual marks. Marked areas, where Streptomycetaceae dominated, exhibited much lower diversity compared with unmarked zones, where Pseudonocardiaceae were prevalent. Unfortunately, comparisons of retrieved sequences with those in public databases yield low similarity scores that do not permit species associations to be made; therefore, we cannot hypothesize whether the presence of these OTUs is due to human disturbance. Similar to our study, the group Pseudonocardiaceae has been confirmed as a true cave rock dweller (Zhu et al., 2019). Moreover, this group has been identified in pigmented zones of pristine caves (Lavoie et al., 2017). Therefore, retrieved sequences may correspond to autochthonous species of the caves. In addition, members of the group Pseudonocardiaceae degrade complex molecules (Lavoie et al., 2017; Porca et al., 2012), which suggests that the unmarked areas were not completely oligotrophic (Tomczyk‐Żak & Zielenkiewicz, 2016), perhaps as a result of past treatment with biocides. However, the higher community diversity in unmarked areas compared with marked areas suggests that cooperative relationships prevail, which is typical for oligotrophic cave environments (Tomczyk‐Żak & Zielenkiewicz, 2016). In this study, specific features of visual marks were not considered according to room location and geological substrate, as the number of replicates was not sufficient; therefore, we focused on findings obtained at the scale of all visual marks considered together.

The lower diversity of marked areas might result from the competitive advantage of invading species (Hamm et al., 2017; Van Elsas et al., 2012). In isolated cave systems, invasions are mainly expected from the cave entrance, similar to Altamira Cave, where *Streptomyces* is the most dominant group, especially in stains (Groth et al., 1999). Our study reports the detection of *S. mirabilis*‐like grouping, *S. fulvissimus*, and *S. niveus*, in caves. These species, which were found in stained areas of Lascaux Cave, have not been previously reported as pathogenic, but rather as producers of secondary metabolites with antimicrobial activity, some of which are used for bioremediation (El‐Sayed, 2012; Flinspach et al., 2014; Kominek, 1972; Myronovskyi et al., 2013; Saberi‐Riseh & Moradi‐Pour, 2021; Schütze et al., 2014). Regarding *S. fulvissimus* and *S. niveus*, the OTUs belonging to these species were close to the GenBank sequences obtained from torrent muddy soil (CP071044.1), rhizosphere (CP048397.1), and deep‐sea sediment (CP018047.1). This seemed to confirm the ability of these species to survive in extreme environments away from human activity. Regarding the *S. mirabilis*‐like grouping, the most proportionally abundant species in Lascaux stains found in this study, OTUs were close to the GenBank sequences obtained from fresh creek bank soil (CP074102.1). The set of OTUs associated with this group is positioned in a phylogenetic clade with only 60% bootstrap, which suggests that they could represent a new taxon within *Actinobacteria* communities, producers of stains in Lascaux Cave. Considering the reported antimicrobial activity of *S. mirabilis*, we believe that the ability of this probable new taxon to produce secondary metabolites may assist, through interactions with other microorganisms, in its survival in this stained environment.

Hypothetically, *Streptomyces* might also contribute to pigment production (Abdel‐Haliem et al., 2013; Cuezva et al., 2012), or affect the pigment‐forming fungi in Lascaux Cave (De La Rosa et al., 2017; Frey‐Klett et al., 2011) based on their ability to cooperate with fungi and promote mycelial extension and secondary metabolite production (Frey‐Klett et al., 2011). A previous study revealed the co‐occurrence of *Streptomyces* and pigment‐forming fungi, *Acremonium* and *Exophiala*, in Lascaux Cave, although only in unmarked areas (Alonso et al., 2018). Moreover, *S. mirabilis* is resistant to heavy metals (Schütze et al., 2014); this might play a role in the formation of pigmented marks since protection against toxicity of heavy metals and other chemicals has also been linked to melanin production (Nosanchuk & Casadevall, 2003). Therefore, the role of *Streptomyces* in the development of visual marks should be studied in more detail to evaluate whether this group may represent a missing link in our understanding of stain development in Lascaux Cave.

## 5 CONCLUSION

Despite the limitations of our database, which yielded a high number of unclassified sequences, the *hsp*65 gene demonstrated its utility to complement the 16S rRNA gene which cannot resolve species but encompasses a larger part of the actinobacterial community. The use of *hsp*65 gene allowed us to detect, with a robust bootstrap, several species not previously reported in caves, demonstrating that it can be used reliably to differentiate *Actinobacteria* species in such environments. We expect these limitations to be addressed with the enrollment of new *Actinobacteria* sequences. This can be performed periodically by requesting other public databases, such as BLAST, or by enrolling sequences of previously identified strains isolated from environmental studies. The *Actinobacteria* community at different spatial levels reflected the natural quality of the caves and different locations within Lascaux Cave. Anthropization was shown to shape *Actinobacteria* communities, which altered typical cave communities and was exhibited by the *Actinobacteria* indicator taxa. Even if rare opportunistic pathogens from the *Actinobacteria* phylum were detected in anthropized caves, it is unknown whether these detected microorganisms represent an important health risk for visitors. We also found that marked areas on the surfaces of Lascaux Cave are important for shaping *Actinobacteria* communities, with particular reference to *Streptomyces* species, whose role in the formation of visual marks requires further research. Further studies are needed to isolate the *Actinobacteria* taxa detected in these caves to determine the true infectious risk associated with these pathogens and their role in the pigmentation of the marked areas. A better understanding of *Actinobacteria* functioning in caves will be important to guide efforts in the conservation of Paleolithic cave heritage.

## AUTHOR CONTRIBUTIONS

*Data curation‐lead, formal analysis‐lead, investigation‐equal, methodology‐lead, and writing—original draft‐lead*: **Andrea Buresova**. *Formal analysis‐equal and software‐lead*: **Jan Kopecky**. *Investigation‐equal, validation‐equal, and writing—review and editing‐equal*: **Marketa Sagova‐Mareckova**. *Investigation‐supporting and resources‐equal*: **Lise Alonso**. *Data curation‐equal, investigation‐supporting, methodology‐supporting, and software‐supporting*: **Florian Vautrin**. *Conceptualization‐lead, funding acquisition‐lead, investigation‐equal, resources‐lead, and writing—review and editing‐equal*: **Yvan Moënne‐Loccoz**. *Conceptualization‐lead, funding acquisition‐lead, investigation‐lead, methodology‐lead, supervision‐lead, validation‐lead, and writing—review and editing‐lead*: **Veronica Rodriguez Nava**.

## CONFLICTS OF INTEREST

None declared.

## ETHICS STATEMENT

None required.

## Data Availability

The Illumina MiSeq amplicon sequences have been deposited in the NCBI Sequence Read Archive under BioProject PRJNA695576 (*hsp*65; https://www.ncbi.nlm.nih.gov/bioproject/PRJNA695576) and PRJNA694921 (16S rRNA gene; https://www.ncbi.nlm.nih.gov/bioproject/PRJNA694921). The Supplementary Table is available in Zenodo at https://doi.org/10.5281/zenodo.6312516 (*hsp*65 OTUs, cutoff 99%—their inferred taxonomic allocations according to the *hsp*65 database and, for selected OTUs, closest species obtained from GenBank BLAST with percent identity). The *hsp*65 metabarcoding DNA sequence database for taxonomic allocations using Mothur is available in Zenodo at https://doi.org/10.5281/zenodo.5576073.

## 1

See Figure A1, A2, A3, A4, A5, A6, A7, A8.

See Table A1, A2, A3, A4, A5, A6, A7.

**Table A1:** Location of cave samples and list of gene markers sequenced using Illumina MiSeq

Sample ID	Sample number	Cave name	Room	Area	Obtained sequences
Apse‐S1	La568	Lascaux	Apse		16S rRNA gene
Apse‐S2	La572	Lascaux	Apse	Visual mark	*hsp*65, 16S rRNA gene
Apse‐S3	La574	Lascaux	Apse		*hsp*65, 16S rRNA gene
Apse‐U1	La479	Lascaux	Apse		*hsp*65, 16S rRNA gene
Apse‐U2	La480	Lascaux	Apse	Unmarked area	*hsp*65, 16S rRNA gene
Apse‐U3	La481	Lascaux	Apse		*hsp*65, 16S rRNA gene
Apse‐DZ1	La464	Lascaux	Apse		16S rRNA gene
Apse‐DZ2	La466	Lascaux	Apse		*hsp*65
Apse‐DZ3	La468	Lascaux	Apse	Visual mark	*hsp*65, 16S rRNA gene
Apse‐DZ4	La470	Lascaux	Apse		16S rRNA gene
Apse‐DZ5	La472	Lascaux	Apse		*hsp*65, 16S rRNA gene
Apse‐DZ6	La474	Lascaux	Apse		*hsp*65, 16S rRNA gene
Apse‐UDZ1	La583	Lascaux	Apse		16S rRNA gene
Apse‐UDZ2	La584	Lascaux	Apse		16S rRNA gene
Apse‐UDZ3	La585	Lascaux	Apse	Unmarked area	*hsp*65, 16S rRNA gene
Apse‐UDZ4	La589	Lascaux	Apse		*hsp*65, 16S rRNA gene
Apse‐UDZ5	La590	Lascaux	Apse		16S rRNA gene
Apse‐UDZ6	La591	Lascaux	Apse		*hsp*65, 16S rRNA gene
Dia‐S1	La440	Lascaux	Diaclase		*hsp*65, 16S rRNA gene
Dia‐S2	La443	Lascaux	Diaclase	Visual mark	*hsp*65, 16S rRNA gene
Dia‐S3	La444	Lascaux	Diaclase		16S rRNA gene
Dia‐U1	La433	Lascaux	Diaclase		*hsp*65, 16S rRNA gene
Dia‐U2	La434	Lascaux	Diaclase	Unmarked area	*hsp*65, 16S rRNA gene
Dia‐U3	La435	Lascaux	Diaclase		*hsp*65, 16S rRNA gene
Passage B‐S1	La457	Lascaux	Passage banks		*hsp*65, 16S rRNA gene
Passage B‐S2	La458	Lascaux	Passage banks	Visual mark	*hsp*65, 16S rRNA gene
Passage B‐S3	La459	Lascaux	Passage banks		*hsp*65, 16S rRNA gene
Passage B‐U1	La445	Lascaux	Passage banks		*hsp*65, 16S rRNA gene
Passage B‐U2	La446	Lascaux	Passage banks	Unmarked area	*hsp*65, 16S rRNA gene
Passage B‐U3	La447	Lascaux	Passage banks		*hsp*65, 16S rRNA gene
Passage IP‐S1	La555	Lascaux	Passage inclined planes		*hsp*65, 16S rRNA gene
Passage IP‐S2	La556	Lascaux	Passage inclined planes	Visual mark	*hsp*65, 16S rRNA gene
Passage IP‐S3	La558	Lascaux	Passage inclined planes		*hsp*65, 16S rRNA gene
Passage IP‐U1	La549	Lascaux	Passage inclined planes		*hsp*65, 16S rRNA gene
Passage IP‐U2	La550	Lascaux	Passage inclined planes	Unmarked area	*hsp*65, 16S rRNA gene
Passage IP‐U3	La551	Lascaux	Passage inclined planes		16S rRNA gene
SAS‐1‐S1	La530	Lascaux	Sas‐1 (airlock‐1 entrance zone, compartment 2)		*hsp*65, 16S rRNA gene
SAS‐1‐S2	La531	Lascaux	Sas‐1 (airlock‐1 entrance zone, compartment 2)	Visual mark	*hsp*65, 16S rRNA gene
SAS‐1‐S3	La532	Lascaux	Sas‐1 (airlock‐1 entrance zone, compartment 2)		*hsp*65, 16S rRNA gene
SAS‐U1	La542	Lascaux	Sas‐1 (airlock‐1 entrance zone, compartment 2)	Unmarked area	*hsp*65
SAS‐U3	La544	Lascaux	Sas‐1 (airlock‐1 entrance zone, compartment 2)		*hsp*65
Rouf1	Ro10	Rouffignac			*hsp*65, 16S rRNA gene
Rouf2	Ro11	Rouffignac			*hsp*65
Rouf3	Ro12	Rouffignac	x	x	*hsp*65
Rouf4	Ro13	Rouffignac			*hsp*65, 16S rRNA gene
Rouf5	Ro14	Rouffignac			*hsp*65, 16S rRNA gene
Rouf6	Ro15	Rouffignac			16S rRNA gene
Mouf1	Mf20	Mouflon			16S rRNA gene
Mouf2	Mf22	Mouflon			*hsp*65, 16S rRNA gene
Mouf3	Mf23	Mouflon	x	x	*hsp*65
Mouf4	Mf24	Mouflon			*hsp*65, 16S rRNA gene
Mouf5	Mf25	Mouflon			*hsp*65
Mouf6	Mf26	Mouflon			16S rRNA gene
Reil1	Re10	Reille			*hsp*65
Reil2	Re11	Reille			16S rRNA gene
Reil3	Re13	Reille	x	x	*hsp*65, 16S rRNA gene
Reil4	Re9	Reille			*hsp*65, 16S rRNA gene
Reil5	Re14	Reille			16S rRNA gene

**Table A2:** Genomes of *Actinobacteria* reference strains from the orders Streptomycetales and Corynebacteriales retrieved from the Integrated Microbial Genome and Microbiome database and used for in silico analyses: comparison of the *hsp*65 and 16S rRNA gene partial sequences

Order	Family	Genus	Species	Order	Family	Genus	Species
Corynebacteriales	Corynebacteriaceae	*Corynebacterium*	*Corynebacterium ammoniagenes* KCCM 40472	Streptomycetales	Streptomycetaceae	*Streptomyces*	*Streptomyces agglomeratus* 37742
			*Corynebacterium atypicum* R2070				*Streptomyces albulus* NK660
			*Corynebacterium aurimucosum* ATCC 700975				*Streptomyces albus* J1074
			*Corynebacterium callunae* DSM 20147				*Streptomyces autolyticus* CGMCC0516
			*Corynebacterium camporealensis* DSM 44610				*Streptomyces avermitilis* MA‐4680
			*Corynebacterium crudilactis* JZ16				*Streptomyces bingchenggensis* BCW‐1
			*Corynebacterium diphtheriae* PW8				*Streptomyces cattleya* DSM 46488
			*Corynebacterium glutamicum* CP				*Streptomyces cattleya* NRRL 8057
			*Corynebacterium pseudotubrculosis* 1002B				*Streptomyces clavuligerus* F613‐1
			*Corynebacterium pseudotubrculosis* MB14				*Streptomyces coelicolor* A3(2)
			*Corynebacterium ulcerans* 131001				*Streptomyces collinus* Tu 365
			*Corynebacterium xerosis* DSM 20743				*Streptomyces flavogriseus* IAF 45
							*Streptomyces formicae* KY5
	Gordoniaceae	*Gordonia*	*Gordonia polyisoprenivorans* VH2 DSM 44266				*Streptomyces fulvissimus* DSM40593
			*Gordonia* sp. 1D				*Streptomyces globisporus* C‐1027
			*Gordonia* sp. KTR9				*Streptomyces griseus* susp. griseus NBRC13350
			*Gordonia* sp. QH‐11				*Streptomyces hygroscopicus jinggangensis* 5008
			*Gordonia* sp. YC‐JH1				*Streptomyces hygroscopicus jinggangensis* TL01
			*Gordonia terrae* 3612				*Streptomyces katrae* S3/4
							*Streptomyces leewenhoekii* DSM 42122
	Mycobacteriaceae	*Mycobacterium*	*Mycobacterium avium* 2285 (R)				*Streptomyces lincolensis* NRRL 2936
			*Mycobacterium avium* paratuberculosis E1				*Streptomyces lividans* TK24
			*Mycobacterium bovis* 1595				*Streptomyces lunaelactis* MM109
			*Mycobacterium canettii* CIPT 140070008				*Streptomyces lydicus* A02
			*Mycobacterium indicus pranii* MTCC 9506				*Streptomyces lydicus* GS93/23
			*Mycobacterium intracellulare* 1956				*Streptomyces malaysiensis* DSM 4137
			*Mycobacterium kansasii* 662				*Streptomyces niveus* SCSIO 3406
			*Mycobacterium lepraemurium* Hawaii				*Streptomyces pactum* KLBMP 5084
			*Mycobacterium leprae* TN NC 002677				*Streptomyces parvulus* 2297
			*Mycobacterium liflandii* 128FXT				*Streptomyces pluripotens* MUSC 137
			*Mycobacterium marinum* M				*Streptomyces pristinaespiralis* HCCB 10218
			*Mycobacterium marseillense* FLAC0026				*Streptomyces puniciscabiei* TW1S1
			*Mycobacterium shigaense* UN‐152				*Streptomyces purpureus* KA281
			*Mycobacterium simiae* MO323				*Streptomyces reticuli* TUE45
			*Mycobacterium tuberculosis* 0A092DS				*Streptomyces sampsonii* KJ40
			*Mycobacterium tuberculosis* 26105				*Streptomyces scabiei* 87.22
							*Streptomyces* sp. PAMC26508
	Nocardiaceae	*Nocardia*	*Nocardia brasiliensis* ATCC 700358				*Streptomyces* sp. S10(2016)
			*Nocardia brasiliensis* FDAARGOS 352				*Streptomyces* sp. SirexAA‐E
			*Nocardia cyriacigeorgica* MDA3349				*Streptomyces* sp. Tu6071
			*Nocardia farcinica* IFM 10152				*Streptomyces* sp. XZHG99
			*Nocardia nova* SH22a				*Streptomyces venezuelae* Shinobu 719
			*Nocardia seriolae* EM150506				*Streptomyces violaceusniger* Tu 4113
			*Nocardia seriolae* UTF1				*Streptomyces xiamenensis* 318
			*Nocardia terpenica* NC YFY NT001				*Streptomyces xiamenensis* MCCC1A01550
							*Streptomyces xinghaiensis* S187
	Nocardiaceae	*Rhodococcus*	*Rhodococcus aetherivorans* IcdP1				
			*Rhodococcus coprophilus* NCTC 10994			*Kitakatospora*	*Kitakatospora setae* KM‐6054
			*Rhodococcus equi* 103S				
			*Rhodococcus erythropolis* CCM2595				
			*Rhodococcus erythropolis* PR4				
			*Rhodococcus fascians* D188				
			*Rhodococcus hoagii* DSSKP‐R‐001				
			*Rhodococcus opacus* B4				
			*Rhodococcus opacus* PD630				
			*Rhodococcus pyridinivorans* SB3094				
			*Rhodococcus qingshengii* djl‐6‐2				
			*Rhodococcus rhodochrous* NCTC 10210				
			*Rhodococcus ruber* P14				
			*Rhodococcus* sp. BCP1				
			*Rhodococcus* sp. RHA1				

**Table A3:** Percentage complementarity between the *hsp*65‐specific forward (TB11) and reverse (TB12) primers and the *hsp*65 sequence database (*hsp*65 sequences and GROESL sequences, paralogs to *hsp*65) with respect to the number of mismatches

	Number of mismatches	Match to *hsp*65 (%)	Match to GROESL (%)
Forward primer	0	30.8	0
	1	74.8	0
	2	86	4.2
	3	98.1	56.2
	4	99.1	85.4
	5	100	100
Reverse primer	0	6.5	0
	1	44.9	0
	2	89.7	52.1
	3	96.3	64.6
	4	100	81.2
	5	100	89.6

**Table A4:** Percent coverage of primers to the *Actinobacteria* RefSeq representative genomes from NCBI of different classes and orders

Class	Order	Number of RefSeq representative genomes	Percentual primer matches
Acidimicrobiia		8	38
Coriobacteriia		63	10
Nitriliruptoria		5	0
Rubrobacteria		7	43
Thermoleophilia		7	14
Actinobacteria			
	Acidothermales	1	0
	Actinomycetales	75	27
	Actinopolysporales	8	100
	Bifidobacteriales	87	68
	Catenulisporales	3	100
	Corynebacteriales	473	83
	Frankiales	18	94
	Geodermatophilales	34	100
	Glycomycetales	13	77
	Kineosporiales	5	80
	Micrococcales	482	53
	Micromonosporales	106	100
	Nakamurellales	5	20
	Propionibacteriales	120	63
	Pseudonocardiales	151	74
	Streptomycetales	348	95
	Streptosporangiales	112	88

*Note*: Only matches with no mismatches on the last three nucleotides of the 3′ end and no more than four overall mismatches were included.

**Table A5:** The number of *Actinobacteria* species identified by the *hsp*65 marker for each respective genus

Family	Genus (number of recovered species)			
Nocardiaceae	*Nocardia* (24)	*Rhodococcus* (10)		
Streptomycetaceae	*Streptomyces* (30)	*Kitasatospora* (1)		
Mycobacteriaceae	*Mycobacterium* (26)			
Pseudonocardiaceae	*Amycolatopsis* (7)	*Pseudonocardia* (4)	*Lentzea* (2)	*Actinomycetospora* (1)
	*Kibdelosporangium* (1)	*Thermocrispum* (1)	*Saccharothrix* (1)	*Alloactinosynnema* (1)
	*Sciscionella* (1)	*Actinoalloteichus* (1)	*Actinokineospora* (1)	
Norcardioidaceae	*Nocardioides* (6)	*Kribbella* (2)	*Pimelobacter* (1)	
	*Marmoricola* (1)	*Aeromicrobium* (1)		
Streptosporangiaceae	*Microbispora* (1)	*Microtetraspora* (1)	*Nonomuraea* (1)	*Planomonospora* (1)
	*Sinosporangium* (1)	*Streptosporangium* (1)		
Micromonosporaceae	*Micromonospora* (3)	*Longispora* (1)	*Catelliglobosispora* (1)	*Plantactinospora* (1)
Intrasporangiaceae	*Janibacter* (3)	*Knoellia* (1)		
Actinomycetaceae	*Actinomyces* (3)			
Jiangellaceae	*Jaingella* (3)			
Microbacteriaceae	*Agromyces* (1)	*Microcella* (1)		
Propionibacteriaceae	*Cutibacterium* (1)	*Propionibacterium* (1)		
Geodermatophilaceae	*Geodermatophilus* (1)	*Modestobacter* (1)		
Micrococcaceae	*Micrococcus* (1)	*Rothia* (1)		
Dermabacteraceae	*Brachybacterium* (2)			
Brevibacteriaceae	*Brevibacterium* (2)			
Corynebacteriaceae	*Corynebacterium* (2)			
Catenulisporaceae	*Catenulispora* (1)			
Dermacoccaceae	*Kytococcus* (1)			
Actinobacteria_incertae_sedis	*Thermobispora* (1)			
Nakamurellaceae	*Nakamurella* (1)			
Sporichthyaceae	*Sporichthya* (1)			
Nocardiopsaceae	*Thermobifida* (1)			

**Table A6:** *t* and *F* tests to compare means and variation of diversity indices for 37 common samples of *hsp*65 and 16S rRNA gene markers at 97% and 99% OTUs cutoffs

			*t* Test		*F* test	
	*hsp*65	16S rRNA	*t*	*p* Value	*F*	*p* Value
99% OTU						
Chao‐1	260.78 ± 137.05	182.17 ± 82.02	2.98	0.003	2.75	0.017
Simpson evenness	0.29 ± 0.25	0.089 ± 0.05	4.72	<0.001	26.805	<0.001
Inverse Simpson	6.57 ± 4.97	14.07 ± 6.29	5.66	<0.001	1.6	0.153
97% OTU						
Chao‐1	75.74 ± 42.26	89.12 ± 36.35	1.45	0.15	1.32	0.588
Simpson evenness	0.38 ± 0.26	0.15 ± 0.11	4.91	<0.001	6.28	0.002
Inverse Simpson	4.09 ± 2.74	9.11 ± 4.44	5.82	<0.001	2.64	0.013

*Note*: For each marker, averages and standard deviations are indicated.

Abbreviation: OTU, operational taxonomic unit.

**Table A7:** Differences between *Actinobacteria* communities between (A) caves, (B) different locations within Lascaux Cave, and (C) marked (S) and unmarked (U) areas calculated by analysis of molecular variance (AMOVA) and homogeneity of molecular variance (HOMOVA) for the *hsp*65 and 16S rRNA gene markers (97% OTUs cutoff). Degrees of freedom (*df*), F ‐ statistics (*F*) and probability values (*p*).

		Cave								Lascaux Cave location								Surface area		
A. AMOVA		Lascaux		Rouffignac		Reille				Sas‐1		Passage B		Passage IP		Apse			Unmarked	
***hsp*** **65**		*df*	*F*, *p*	*df*	*F*, *p*	*df*	*F*, *p*			*df*	*F*, *p*	*df*	*F*, *p*	*df*	*F*, *p*	*df*	*F*, *p*		*df*	*F*, *p*
	Rouffignac	*13*	5.72***						Passage B	*10*	7.858**							Marked	32	*2*.23**
	Reille	*11*	3.614**	*7*	4.617				Passage IP	*9*	3.978	10	4.419**							
	Mouflon	*12*	6.265**	*8*	8.033*	*6*	4.736		Apse	*16*	4.505***	17	5.903***	16	2.572***					
									Diaclase	*9*	4.518	10	5.635**	*9*	2.786	16	3.193***			
	Overall	20	5.317***						Overall	32	4.279***									

16S rRNA		**Lascaux**		**Rouffignac**		**Reille**				**Sas‐1**		**Passage B**		**Passage IP**		**Apse**			**Unmarked**	
		***df***	***F*, *p***	***df***	***F*, *p***	***df***	***F*, *p***			***df***	***F*, *p***	***df***	***F*, *p***	***df***	***F*, *p***	***df***	***F*, *p***		***df***	***F*, *p***
	Rouffignac	*15*	5.79***						Passage B	*8*	10.063							Marked	37	2.66***
	Reille	*15*	5.43***	*7*	6.263				Passage IP	*8*	4.863	11	9.013**							
	Mouflon	*15*	5.36**	*7*	6.329	*7*	2.260		Apse	*19*	4.09***	*22*	8.115***	*22*	2.788**					
									Diaclase	*8*	2.723	11	5.79**	11	2.902**	22	3.005***			
	Overall	*23*	5.274***						Overall	37	4.656***									

	Bonferonni pair‐wise err. rate: 0.008								Bonferonni pairwise err. rate: 0.005									Exp.‐wise err. rate: 0.05		
	0.008 > **p* > 0.005 > ***p* > 0.001 > ****p*								0.005 > **p* > 0.0025 > ***p* > 0.001 > ****p*									0.05 > **p* > 0.01 > ***p* > 0.001 > ****p*		

B. HOMOVA	**Cave**						**Lascaux Cave location**					**Surface area**								
*hsp*65		***B*, *p***					***B*, *p***	***B*, *p***					***B*, *p***							
		**Rouffignac**	**Lascaux**	**Reille**			**Sas‐1**	**Passage B**	**Passage IP**	**Apse**			Unmarked (0.405)							
	Rouffignac (0.193)					Sas‐1 (0.229)						Marked (0.453)	0.048*							
	Lascaux (0.303)	0.235*				Passage B (0.212)	0.006													
	Reille (0.342)	0.193	0.010			Passage IP (0.388)	0.246	0.367												
	Mouflon (0.160)	0.026	0.359	0.295		Apse (0.364)	0.270*	0.433*	0.005											
					Diaclase (0.333)	0.126	0.206	0.02	0.010										

	Overall	0.573*					Overall	0.693*											
16S rRNA		***B*, *p***					***B*, *p***						***B*, *p***							
		**Rouffignac**	**Lascaux**	**Reille**			**Sas‐1**	**Passage B**	**Passage IP**	**Apse**			Unmarked (0.354)							
	Rouffignac (0.145)					Sas‐1 (0.153)						Marked (0.405)	0.0786*							
	Lascaux (0.276)	0.380				Passage B (0.133)	0.012													
	Reille (0.23)	0.133	0.034			Passage IP (0.133)	0.166	0.531*												
	Mouflon (0.199)	0.062	0.105	0.014		Apse (0.328)	0.364	1.227*	0.079											
						Diaclase (0.374)	0.415	1.166*	0.134*	0.031										
		Overall	0.432*					Overall	1.689*											
	Bonferonni pairwise err. rate: 0.008 > **P*					Bonferonni pairwise err. rate: 0.005 > **P*						Exp.‐wise err. rate: 0.05 > **P*								

Abbreviation: *df*, degrees of freedom; *F*, F‐statistics; OTU, operational taxonomic unit; *p*, probability values.
